# Current practices and challenges in the management of cancer-associated thrombosis: a survey of Italian oncologists

**DOI:** 10.3389/fonc.2025.1579464

**Published:** 2025-08-06

**Authors:** Andrea Antonuzzo, Paola Ermacora, Gaetano Lanzetta, Maurizio Lucchesi, Marco Platania, Paolo Bossi

**Affiliations:** ^1^ Medical Oncology Department, Fondazione IRCCS Istituto Nazionale dei Tumori di Milano, Milan, Italy; ^2^ Oncology department, Presidio Ospedaliero S. Maria della Misericordia di Udine, Udine, Italy; ^3^ Medical Oncology Unit , Italian Neuro-Traumatology Institute, Grottaferrata, Italy; ^4^ U.O. Oncologia Medica delle Apuane, Azienda USL Toscana Nord Ovest, Pisa, Italy; ^5^ Department of Biomedical Sciences, Humanitas University, Milan, Italy; ^6^ IRCCS Humanitas Research Hospital, Milan, Italy

**Keywords:** cancer-associated thrombosis, venous thromboembolism, thromboprophylaxis in oncology, direct oral anticoagulants, thrombosis management

## Abstract

**Purpose:**

This study investigates current practices and challenges in managing cancer-associated thrombosis (CAT) among Italian oncologists, with the objective of evaluating adherence to guidelines for primary thromboprophylaxis, treatment approaches, and safety considerations. Additionally, it aims to identify areas for potential improvement in clinical decision-making and standardization of CAT management.

**Methods:**

A cross-sectional survey was conducted between February and June 2024 among Italian oncologists, facilitated by the Italian Network for Supportive Care in Oncology (NICSO). The online survey comprised 16 multiple-choice questions that addressed primary thromboprophylaxis practices, thrombosis treatment, and anticoagulation safety concerns.

**Results:**

A total of 75 oncologists, evenly distributed across the Italian territory, participated in the survey. Among them, 48% consistently administered primary thromboprophylaxis, with a higher prevalence observed in cases of pancreatic (64%) and lung cancers (12%). Overall, 61% utilized risk assessment models (RAMs), mainly the Khorana score. Drug preference varied, with 89% favoring low-molecular-weight heparin (LMWH) for thromboprophylaxis. For established thrombosis, 72% preferred LMWH, administering treatment to 52% of patients for 3 to 6 months. Awareness of drug-drug interactions was high (93%), and 83% expressed concerns about bleeding risks, with renal impairment identified as a significant comorbidity.

**Conclusion:**

The survey highlights variability in CAT management, with limited use of RAMs and personalized treatment plans. These findings underscore the need for enhanced clinician education and standardized guidelines to optimize CAT management, including strategies to address bleeding risk and improve the safety of anticoagulation therapy.

## 1 Introduction

Cancer-associated thrombosis (CAT) is an increasingly significant cause of morbidity and mortality in patients with cancer (1, 2). Venous thromboembolism (VTE), which includes deep vein thrombosis and pulmonary embolism, affects approximately 5% to 20% of cancer patients, depending on the tumor type and treatment modalities (3).

The pathogenesis of CAT is multifactorial, involving elements of Virchow’s triad: stasis, endothelial injury, and hypercoagulability. In cancer patients, tumor cells release procoagulant factors, such as tissue factor and cancer procoagulant, which initiate and propagate the clotting cascade (4, 5). Furthermore, chemotherapy and radiotherapy exacerbate the thrombosis risk by damaging the vascular endothelium and increasing pro-inflammatory cytokines. Patient-related factors, including immobility and advanced age, also contribute to the elevated thrombosis risk (4, 5). Accordingly, the 6-month VTE risk in patients with cancer is up to 12-fold higher than in the general population and up to 23-fold higher in patients receiving chemotherapy or targeted therapy (6). High rates of thromboembolic events have also been reported in cancer patients treated with immunotherapy, although a causal effect has not yet been established (7, 8).

Current guidelines strongly recommend pharmacologic primary prophylaxis for CAT in hospitalized surgical and medical cancer patients and emphasize its potential benefits for select ambulatory patients (9–13). Despite this evidence, CAT remains an underrecognized issue in cancer patients, with prophylaxis utilization still suboptimal in many cases (14, 15). Moreover, CAT management is highly variable in real-world practice, reflecting the complexity of individualized patient care (16, 17). Indeed, several factors, such as cancer type, patient comorbidities, and the use of risk assessment models (RAMs), may influence the decision to implement thromboprophylaxis. In particular, one of the primary challenges in CAT management is the elevated risk of bleeding, compounded by potential drug–drug interactions (DDIs) between anticoagulants and anticancer therapies, which complicate treatment decisions and affect patient outcomes (18). Additionally, cancer patients face an increased risk of bleeding due to factors, such as thrombocytopenia and the frequent use of nephrotoxic or hepatotoxic agents, making the selection of suitable anticoagulation strategies particularly challenging. Comorbid conditions, such as renal impairment, which are prevalent in this population, further complicate the choice of anticoagulation therapy, requiring a careful balance between the benefits and risks of treatment (19). Several validated RAMs, such as the Khorana and PROTECHT scores, have been developed to identify cancer patients at elevated risk of venous thromboembolism (VTE), particularly in the ambulatory setting. These tools consider tumor type, platelet count, hemoglobin level, leukocyte count, and body mass index. Certain malignancies are associated with a particularly high incidence of VTE, and are often weighted heavily in these models. Despite their utility, RAMs remain underutilized in routine clinical practice, contributing to inconsistencies in thromboprophylaxis decisions.

This survey study aims to investigate current clinical practices regarding primary thromboprophylaxis, thrombosis treatment, and safety considerations in the Italian scenario, identifying areas of consensus and deviation in oncologic care settings. These findings are intended to guide future standardization efforts​ and focus improvement actions in this healthcare area.

## 2 Methods

A cross-sectional survey was conducted to assess the current clinical practices and perceptions of oncologists regarding the management of CAT. The survey was distributed online with the support of the Italian Network for Supportive Care in Oncology (NICSO) board of directors between February and June 2024. The questionnaire consisted of 16 multiple-choice questions divided into three sections (see Appendix I for the full text of the survey):

1. Primary thromboprophylaxis practices;
2. Treatment of established thrombosis;
3. Safety considerations related to anticoagulation therapy.

The survey examined key aspects such as the use of thromboprophylaxis, preferred treatment regimens, use of RAMs, awareness of DDIs, and safety concerns, including bleeding and renal impairment. Data were collected anonymously through SurveyMonkey software and analyzed descriptively, with the results presented as frequencies and percentages of total respondents.

## 3 Results

**Figure 1:**
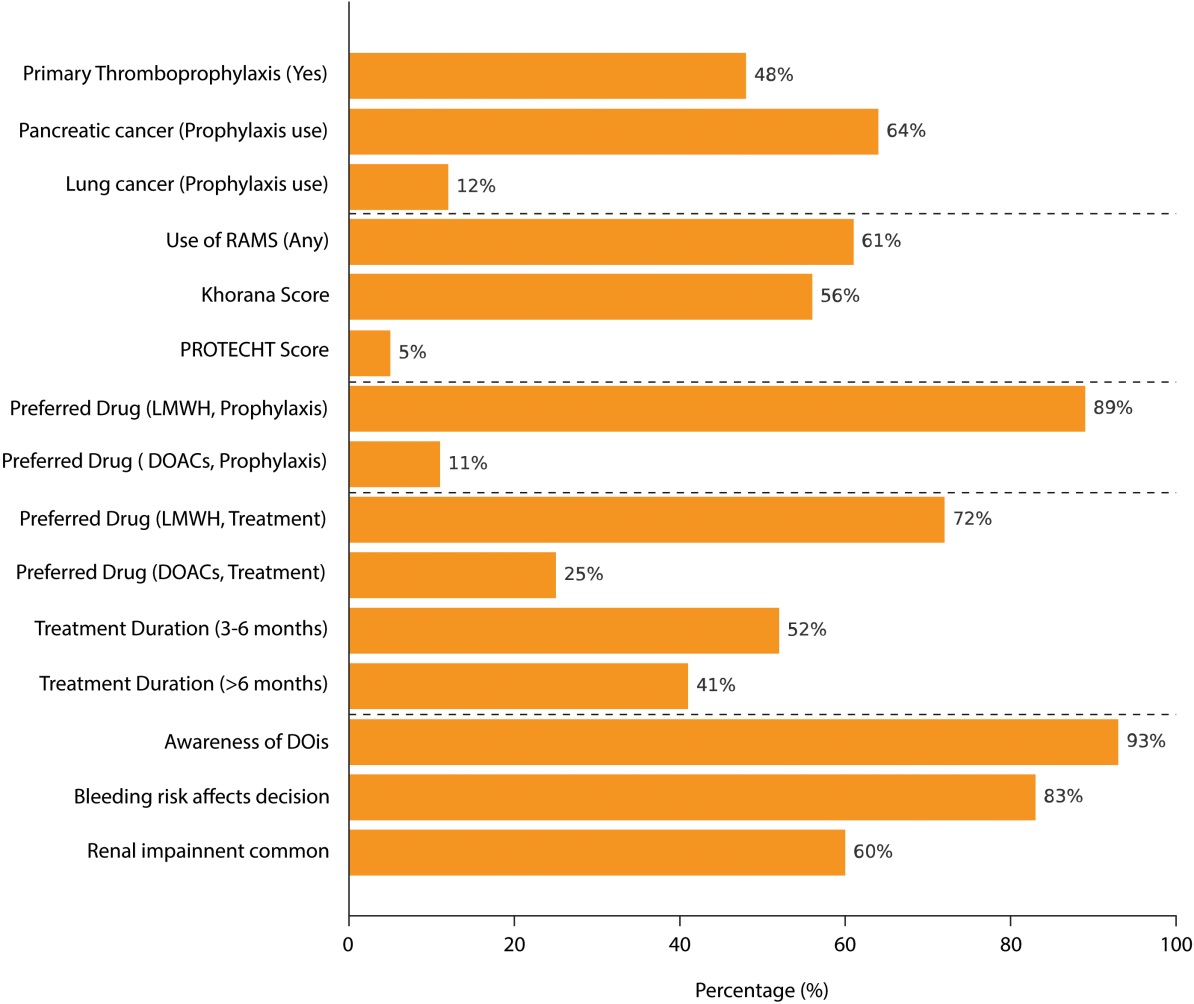
Summary of Survey Findings on CAT Management.

A total of 75 oncologists, evenly distributed across the Italian territory, participated in the survey. **Figure 1** summarizes the main survey results.

### 3.1 Primary thromboprophylaxis

Survey results showed that primary thromboprophylaxis is routinely administered in cancer patients by 48% (36/75) of oncologists. Pancreatic (64%, 48/75) and lung (12%, 9/75) cancers were the most frequently considered cancer types for thromboprophylaxis. Overall, 61% (46/75) of participants reported using RAM to evaluate thrombosis risk in their patients, i.e., the Khorana score used in 56% (42/75) of cases or the PROTECHT (PROphylaxis of ThromboEmbolism during CHemoTherapy) score in 5% (4/75) of cases. The duration of primary thromboprophylaxis varied, with 44% (33/75) of oncologists administering it during the active cancer phase and 32% (24/75) limiting its use to the hospitalization period. Regarding drug preference for thromboprophylaxis, 89% (67/75) favored low-molecular-weight heparin (LMWH), while 11% (8/75) preferred direct oral anticoagulants (DOACs). Furthermore, 64% (48/75) of oncologists stated that they personally chose the thromboprophylaxis treatment approach for their cancer patients.

### 3.2 Treatment

Overall, 48% (36/75) of oncologists reported fewer than five thrombosis events in a 1-month period among their patients, while 45% (34/75) reported between five and 10 events in the same timeframe. The majority of respondents indicated that they were treating thrombotic events for varying durations. Specifically, 52% (39/75) treated thrombosis for 3–6 months, whereas 41% (31/75) extended treatment beyond 6 months. Regarding treatment choice, 72% (54/75) of oncologists preferred LMWH, while 25% (19/75) selected DOACs. Importantly, 55% (41/75) of oncologists reported being primarily responsible for selecting the type of drug used for thrombosis treatment in their patients.

### 3.3 Safety

A high level of awareness regarding DDIs between anticoagulants and anticancer therapies was reported by 93% (70/75) of respondents, with 95% (71/75) considering DDIs a relevant factor in the selection of anticoagulation treatment. Bleeding risk was also a significant concern, with 83% (62/75) of oncologists indicating that bleeding risk could deter them from prescribing thromboprophylaxis. Additionally, 60% (45/75) of respondents reported renal impairment as a common condition in their cancer patient population. When evaluating safety risks, 68% (51/75) of oncologists personally assessed the risk of bleeding, while 76% (57/75) took responsibility for evaluating the potential risk of DDIs in their patients.

## 4 Discussion

The management of CAT remains a significant challenge because of the complex interplay between cancer progression, the tumor-induced hypercoagulable state, and the increased risk of both thrombosis and bleeding (18).

The findings of this survey study, conducted among 75 Italian oncologists, highlight that only a minority of participants (48%) provided thromboprophylaxis, revealing variability in real-life practices regarding the type and duration of treatment. Several factors may contribute to the reported relatively low rate of thromboprophylaxis. Oncologists often express concerns about bleeding risk, particularly in frail patients or those with comorbidities, which may deter anticoagulant use. In addition, the inconsistent implementation of RAMs, coupled with limited consensus on when to apply prophylaxis in ambulatory settings, may result in clinical inertia. Finally, decision-making processes may vary across institutions and specialties, depending on the degree of involvement from internists, cardiologists, or haematologists, leading to further variability in thromboprophylaxis practices. This variability reflects the ongoing difficulties in balancing efficacy with safety concerns, emphasizing the need for enhanced clinician education, particularly in managing thrombotic risk in more complex clinical scenarios. In particular, to improve adherence to evidence-based CAT management, clinician education should be prioritized across oncology settings. This may include incorporating CAT-focused content into continuing medical education (CME) programs, promoting the routine use of RAMs, and fostering multidisciplinary collaboration with haematologists, cardiologists, and internists. Educational efforts should also address common concerns regarding bleeding risk and drug–drug interactions, enabling oncologists to make more confident and informed decisions about anticoagulant use.

The use of RAMs, such as the Khorana score, is recommended to stratify patients and guide thromboprophylaxis decisions (20). However, consistent with previous evidence, not all participants reported regularly employing these tools, possibly due to variations in patient characteristics or institutional guidelines (21). This highlights the need for improved adoption of updated, disease-specific RAMs, such as the ONKOTEV (ONKOlogie ThrombEmbolie Voraussage) score for pancreatic cancers, given the current limited utilization of available tools (22). Implementing such scores could enable a more precise evaluation of thrombotic risk in oncology patients, leading to better-informed clinical decisions. Accordingly, recent evidence from the literature has shown that implementing a multidisciplinary VTE prevention model in community-based oncology settings has successfully enhanced VTE education, risk assessment, and anticoagulant prophylaxis rates (23).

Concerning the management of CAT, LMWHs have historically been the mainstay for both prophylaxis and treatment (10). However, due to their ease of use and comparable efficacy, DOACs have emerged as an effective alternative, particularly for patients without gastrointestinal cancers (24). Randomized controlled trials have demonstrated the non-inferiority of DOACs over LMWHs in reducing VTE recurrence, with some evidence suggesting a reduced risk of symptomatic VTE (25–29). At the same time, the safety profile of DOACs remains a concern, particularly the increased risk of major bleeding, which is significantly higher in patients with gastrointestinal or genitourinary malignancies (30). The survey results align with these concerns, as most of the participants reported a preference for LMWHs for both VTE prophylaxis (89%) and treatment (72%) (31).

The duration of prophylaxis and treatment varied among respondents. Overall, 44% of oncologists administered prophylaxis during the active cancer phase, while 32% provided it during the hospitalization period. For thrombosis treatment, 52% of oncologists administered therapy for 3–6 months, while 41% extended it beyond 6 months. In most cases, oncologists reported personally choosing the thromboprophylaxis approach (64%) or VTE treatment (55%) for their cancer patients. In other cases, it is likely that decisions involved collaboration with the transfusion medicine department, cardiologists, or internists.

Safety considerations, including the risk of bleeding and potential DDIs, play a critical role in the management of CAT (32). Accordingly, 95% of respondents considered DDIs a relevant factor in selecting anticoagulation treatment, and 83% expressed concerns about bleeding risks as a reason to avoid prophylaxis in certain patients. Safety risk assessments were performed primarily by oncologists (68%), who were also responsible for evaluating the potential risk of DDIs (76%). The introduction of personalized treatment plans based on individual risk factors and comorbidities represents a critical strategy for enhancing clinical outcomes. Furthermore, additional studies are recommended to evaluate the impact of these interactions in clinical practice, with the ultimate goal of refining therapeutic strategies for the safer and more effective management of CAT.

This survey study presents several limitations that should be acknowledged. First, its survey-based design relies on self-reported practices and perceptions, potentially introducing response bias and limiting the generalizability of the findings. Moreover, although geographically distributed, the small sample size of 75 oncologists may not fully capture the diversity of practices across different institutions or countries, limiting external validity. Finally, while the survey explored key aspects of thromboprophylaxis and thrombosis treatment, it did not include detailed patient-level data, which could have provided more granular insights into clinical decision-making processes. At the same time, this was an exploratory survey aimed at capturing real-world practices in a diverse sample of Italian oncologists. Despite the above-mentioned limitations, the study offers valuable real-world insights into current practices and challenges of managing CAT within the Italian scenario. These findings can contribute to the refinement of future guidelines and clinical practices.

## 5 Conclusion

The results of this Italian survey underscore the need for greater clinician awareness and more standardized approaches to CAT management. Particular emphasis should be placed on optimizing RAMs and ensuring the safe, effective use of anticoagulants. Future research should focus on refining guidelines for DOAC use and exploring strategies to mitigate the risks of bleeding and DDIs, ultimately enhancing outcomes for cancer patients at risk of thrombosis.

## Data Availability

The raw data supporting the conclusions of this article will be made available by the authors, without undue reservation.
