# Supplementary material for: Current practices and challenges in the management of cancer-associated thrombosis: a survey of Italian oncologists
Source: Front Oncol. 2025 Aug 6;15:1579464. doi: 10.3389/fonc.2025.1579464 (PMC12364648; doi:10.3389/fonc.2025.1579464)
Supplement: Supplementary file 1 [file DataSheet1.docx]

# Management of cancer-associated thrombosis: insights from clinical practice

SURVEY

# Prophylaxis

1. Do you usually provide thromboprophylaxis in cancer patients?
   1. Yes
   2. No
2. What type of cancer do you usually consider for thromboprophylaxis?
   1. Gastrointestinal cancer
   2. Lung cancer
   3. Pancreatic cancer
   4. Ovarian cancer
   5. None
3. Do you use any risk assessment model (RAM) for the evaluation of the risk of thrombosis in your cancer patients?
   1. Khorana Score
   2. PROTECHT score
   3. None
4. How long do you usually provide thromboprophylaxis?
   1. During the active cancer phase
   2. During the whole cancer trajectory
   3. During the hospitalization period
5. What type of drug do you prefer for thromboprophylaxis in cancer patients?
   1. LMWH
   2. DOAC
   3. Vitamin K antagonist
   4. Whatever drug is available in the hospital
6. Are you, as an oncologist, the one who chooses the treatment approach for thromboprophylaxis in your cancer patients?
   1. Yes
   2. No

# Treatment

1. How many cancer patients experience thrombosis in your daily practice in a 1-month period?
   1. Less than 5
   2. Between 5 and 10
   3. More than 10
2. How long do you treat thrombosis?
   1. Less than 3 months
   2. Between 3 and 6 months
   3. More than 6 months
3. What type of drug do you prefer to treat thrombosis?
   1. LMWH (Low-molecular-weight heparin)
   2. DOAC (direct-acting oral anticoagulants)
   3. Vitamin K antagonist
   4. Whatever drug is available in the hospital
4. Are you, as an oncologist, the one who chooses what type of drug to use for the treatment of thrombosis?
   1. Yes
   2. No

# Safety

1. Are you aware of the possible drug-drug interactions (DDIs) between anticoagulants and anticancer treatment?
   1. Yes
   2. No
2. Do you consider DDIs a relevant aspect for the choice of anticoagulation treatment?
   1. Yes
   2. No
3. Do you consider the risk of bleeding a reason not to provide prophylaxis?
   1. Yes
   2. No
4. Is renal impairment common in your patients?
   1. Yes
   2. No
5. Are you, as an oncologist, the one who evaluates the risk of bleeding?
   1. Yes
   2. No
6. Are you, as an oncologist, the one who evaluates the risk of DDIs?
   1. Yes
   2. No
